# Supplementary material for: Complexome profiling of the Chlamydomonas psb28 mutant reveals TEF5 as an early PSII assembly factor
Source: Plant Cell. 2025 Mar 17;37(6):koaf055. doi: 10.1093/plcell/koaf055 (PMC12164590; doi:10.1093/plcell/koaf055)
Supplement: koaf055_Supplementary_Data [file koaf055_supplementary_data.zip › psb28_interactive.zip!/plots/intensity/qualityPlots/peptides/UCP1_Cre06.g278750.t1.2.html]

### Cre06.g278750.t1.2

Trivial name: UCP1  
SeqCoverage [median]: 39.90  
Mass: 31.39 kDa  
  
 

| Sequence | MissedCleavage | Mass [Da] | wtIntensity | muIntensity | Score |
| --- | --- | --- | --- | --- | --- |
| GLGPNIAR | 0 | 796.46 | 4033.74 | 2184.94 | 100.07 |
| GMLGTVATIAR | 0 | 1088.60 | 0.00 | 2033.05 | 95.50 |
| IAAGLTTGALGISVASPTDLVK | 0 | 2054.16 | 888.34 | 0.00 | 73.91 |
| IGLYEPVR | 0 | 945.53 | 6367.15 | 5916.28 | 91.04 |
| KYPSAIAAYGIIAR | 1 | 1492.84 | 975.03 | 0.00 | 28.15 |
| LLTPAPSH | 0 | 834.46 | 2487.15 | 1126.23 | 99.92 |
| LQLQAGGNK | 0 | 927.51 | 7445.81 | 4439.57 | 113.50 |
| NAIINAAELASYDQIK | 0 | 1732.90 | 513.06 | 0.00 | 88.19 |
| NEGPLAFYK | 0 | 1037.52 | 4747.64 | 3924.05 | 96.25 |
| VASSSSSQPLSFPR | 0 | 1448.73 | 11190.29 | 8979.94 | 144.70 |
|  |  |  | 38648.2 | 28604.1 |  |
